# Reproduction and the expanding border: pregnant migrants as a ‘problem’ in the 2014 Immigration Act

**DOI:** 10.1177/00380385231157987

**Published:** 2023-04-03

**Authors:** Gwyneth Lonergan

**Affiliations:** Northumbria University

**Keywords:** bordering, citizenship, gender, migration, reproduction

## Abstract

This paper explores the construction of the UK National Health Service (NHS) as a ‘bordering scape’, and the depiction of pregnant migrants as an especial problem, in policy documents and Parliamentary debates around the 2014 Immigration Act. Migrant women’s reproductive practices have long been an object of state anxiety, and a target of state intervention. However, this has been largely overlooked in recent scholarship on the proliferation and multiplication of internal bordering processes. This paper addresses this gap and contributes to conceptualisations of bordering processes as situated and intersectional, arguing that discourses and anxieties around the reproduction of the nation-state play an important role in informing the construction of the proliferating internal border. These discourses and anxieties, which are heavily gendered and racialised, interact with the specificities of individual bordering sites in shaping both bordering processes, and the production of different individuals and groups within these processes.

## Introduction

This paper employs Critical Discourse Analysis to explore the construction of pregnant migrants as an especial ‘problem’ within policy documents and Parliamentary debates discussing NHS bordering within the 2014 Immigration Act. Presently, around the world, bordering processes – which this paper understands as a series of dynamic, contingent ‘practices and discourses that ‘spread’ into the whole of society’ (Paasi, 1999: 670 cited in Mezzadra and Nielsen, 2013: 13) governing entry and settlement into a country – are proliferating and multiplying *within* countries, becoming embedded in a range of sites and institutions, including some critical to reproduction. These processes are *situated* and *intersectional*, in that they are shaped by the site in which they are occurring, and impact individuals differently depending on their social location (Yuval-Davis et al., 2018; 2019). ‘NHS bordering’ refers to the production of bordering processes within and through the UK National Health Service (NHS)^i^, which may place conditions upon, or limit, migrants’ access to NHS services. Building upon, and contributing to, conceptualisations of bordering processes as situated and intersectional, this paper explores the role of gendered and racialised discourses around reproduction in constructing the proliferating internal border. These discourses interact with the specificities of the NHS as a bordering site to produce the ‘pregnant migrant’ as a ‘problem’, and therefore a particular social location, within NHS bordering processes.

Policies around immigration, and those dealing with reproduction, are both about who can be a citizen, and on what terms (Ross, 2006). Immigration policies dictate what requirements must be met to enter a country, settle, and become a citizen. Policies around reproduction – which may include legislation around access to abortion and contraception, policies governing medical care, and welfare state support for families – differentially allocate the resources necessary to exercise reproductive choice, and in doing so indicate who is encouraged, or even expected, to give birth to and/or raise ‘future citizens’, and who is not (Ross, 2006; Solinger, 2001). Migrants are targeted by both kinds of policies; as will be discussed below, bordering processes govern migrants’ access to welfare state services central for reproduction. Indeed, there is a long history of using immigration policy to intervene into, and discipline, migrant women’s reproductive practices, in the UK and elsewhere (Erel, 2018; Lonergan, 2018; Luibhéid, 2013; Solinger, 2013). However, the changing nature of bordering processes, notably their internal multiplication, necessitates a reconceptualization of our understanding of the relationship between immigration policies and reproduction.

The 2014 Immigration Act was first read as a Bill in the UK House of Commons in October 2013, and was debated in both Houses of Parliament before becoming law on 14 May 2014^ii^. It is a major piece of legislation that continues to be one of the key laws governing internal bordering in the UK. The Act significantly expanded internal bordering processes in the UK, enabling the creation of statutory instruments that further entrenched NHS bordering in two main ways. First, through tightening visa checks for secondary care, and introducing sanctions and incentives to ensure that eligible patients were charged for care (Department of Health, 2014); and second, through the introduction of the Immigration Health Surcharge (IHS) on work, family, & student visas^iii^. The legislation permitting visa checks and charging for secondary care had been in place since 1982, and checks had been carried out, albeit in an inconsistent manner, since 2006 (Medien, 2021; Pollock et al., 2005). The IHS was the more novel policy innovation, and served to redefine who could be considered ‘at home’ in the UK.

I begin this paper by discussing welfare bordering through the lens of ‘domopolitics’, in which the nation-state is conceived as a ‘national home’ and migrants as ‘guests’ in need of domesticating (Walters, 2004). Welfare bordering is informed by gendered and racialised discourses around reproduction that indicate who can be trusted to correctly reproduce the ‘national home.’ The existing scholarship on bordering processes as ‘situated and intersectional’ (Yuval-Davis et al., 2018; 2019) is reviewed, with a focus on how this applies to NHS bordering. I then discuss my use of Critical Discourse Analysis to analyse policy papers and parliamentary debates around NHS bordering within the 2014 Immigration Act, before sharing my findings. I argue that policy documents and debates legitimate NHS bordering through calls for migrants to make a ‘fair contribution’ in order to access NHS services. Underpinning this call is a referential strategy naming migrants affected by the IHS as ‘temporary’ rather than ‘ordinary resident’. This marks a significant moment of re-bordering, and serves to associate access to the NHS with being ‘at home’ in the UK. Building on this, I argue that the stigmatisation of pregnant migrants within policy documents and parliamentary debates is a product of the interaction of gendered and racialised discourses around reproduction with the particularities of NHS bordering. As entitlement to NHS services becomes associated with being ‘at home’, pregnant migrants’ access to NHS maternity care is limited both because they are seen as ‘temporary’; and also, paradoxically, because, by having a baby on ‘our NHS’ they are building a family and making a claim to be ‘at home.’ Moreover, the devaluing of reproductive labour under neoliberalism means that pregnant migrants are named within policy documents and debates as especially economically burdensome, a depiction which is perversely legitimatised by the parallel naming of pregnant migrants as a ‘vulnerable group.’

## The welfare state and the reproduction of the ‘national home’

Walters (2004) explores the expansion and securitization of bordering processes through an analytic of *domopolitics*, in which the nation-state is conceived as a ‘national home’, and immigrants as ‘guests’ who must abide by ‘house rules.’ The proliferation of immigration controls is necessary to ‘domesticate’ the ‘national home’, disciplining ‘guests’ and allowing the explusion of the undesirable (Walters, 2004). Welfare state services play a key role. Distinctive conditions on access to the welfare state are central to the construction of the different immigration ‘categories’ into which migrants are sorted (Darling, 2011). Moreover, limiting migrants’ access to the welfare state can serve to encourage them to demonstrate particular behaviours, such as financial self-sufficiency (Anderson, 2013; Lonergan, 2015). In addition to *domesticating* migrants, welfare bordering also sends a message about who belongs in the ‘national home’, by limiting who has access to the resources required to build a home. As Gedalof (2007: 83) argues:

Access to health care, housing and education is an intrinsic part of the processes of cultural reproduction, of making and re-making the place one belongs to. To exclude people from those processes is to say that they are not at home, and that they are not part of that reproductive process.

(Gedalof, 2007).

Through a domopolitical lens, welfare bordering is thus intertwined with the disciplining of migrants’ reproductive practices. Anxieties around national reproductive futures inform welfare bordering processes; differential distribution of access to benefits can serve to indicate who is considered able to reproduce the ‘national home’ and under what circumstances (Lonergan, 2018). Discourses and anxieties around reproduction and welfare bordering are heavily gendered. Under domopolitics, women’s responsibility under (neo)liberal citizenship for reproducing and maintaining the family home (Pateman, 1989) is paralleled by her responsibility for reproducing the ‘national home’ (Lonergan, 2018; Yuval-Davis, 1997). Under (neo)liberalism, both are constituted paradoxically as, critical to the national future and therefore a legitimate site of state intervention; and as an apolitical ‘private’ matter (and therefore not something for which she should expect state support (Lonergan, 2018). As ‘outsiders’, migrant women’s ability to reproduce the national home ‘correctly’ can be especially subject to state and social scrutiny (Coddington, 2020; Lonergan, 2018; Luibhéid, 2013; Marchesi, 2012; Tyler, 2010; Yuval-Davis et al., 2005). Migrant mothers may be viewed as an ‘obstacle’ to their native-born children’s ‘integration’ (Gedalof, 2007; Yuval-Davis et al., 2005). Indeed, because migrant women are seen as potentially incapable of producing future citizens (Cisneros, 2013), their fertility is, in itself, constructed as a potential threat to the nation-state, and a justification for stricter immigration controls (Erel, 2018; Lonergan, 2018; Luibhéid, 2013).

Discourses around the UK ‘national home’ and reproduction are heavily racialised. Successive immigration policies throughout the 1960s and 1970s restricted immigration from the Global South while leaving routes to settlement open to white citizens of the ‘Old Commonwealth’ (Samantrai, 2002; Tyler, 2010). This culminated in the 1981 Nationality Act, which limited *jus soli*^iv^ citizenship to the children of British citizens or permanent residents. Within the context of racialised immigration controls, this reinforced the construction of UK citizenship as associated with whiteness while simultaneously producing Black and Asian British residents, many of whom had been born in (former) British colonies, as a ‘foreign population’ within the state, both ideologically and, sometimes, legally (El-Enany, 2020; Tyler, 2010). The association of Britishness with whiteness, and concomitant racialisation of migration, persists to this day (Bhambra, 2018). Racialised discourses and anxieties around the reproduction of the nation-state were intertwined with the 1981 Act. The ‘right’, and responsibility, of producing the next generation of British citizens was now limited to existing citizens and permanent residents (implicitly racialised as white), while, at the same time, as Tyler (2010: 69) argues ‘maternity wards across Britain [became] ‘border zones’ through which ‘aliens’ enter Britain.’

## Situated intersectionality and NHS bordering

Yuval-Davis et al. (2018; 2019) argue that bordering processes are situated and intersectional. They analyse bordering processes by exploring specific ‘bordering scapes’ – ‘a spatial zone in which specific bordering processes are taking place’ - through multiple individual perspectives that are grounded in social locations produced by mutually constitutive discourses and practices around race, gender, class and other social divisions (Yuval-Davis et al., 2019: 19). They demonstrate how bordering processes not only impact individuals differently depending on their social location, but also shape individual subjectivities and relations between individuals and groups, and influence and are influenced by the bordering sites themselves (Yuval-Davis et al., 2018; 2019). This paper builds on this scholarship, exploring how gendered and racialised discourses around reproduction interact with the specificities of the NHS as a bordering site in constructing situated and intersectional bordering processes, and producing ‘pregnant migrant’ as a particular social location.

While the 2014 Immigration Act significantly expanded NHS bordering, passport checks and charges for overseas visitors’ care were first introduced in 1982 (Medien, 2021). As Medien (2021; 2022) argues, this occurred within the context of the introduction and passage of the 1981 Nationality Act which strengthened the association between Britishness and whiteness (see also El-Enany, 2020; Tyler, 2010). These charges and checks can therefore be read as targeting ethnic minorities, excluding them from a key element of the British welfare state, while subjecting them to surveillance, thereby further racializing the UK ‘national home’. Furthermore, the establishment of the NHS depended upon the wealth built through the British Empire, and migrants from former colonies have historically made up, and continue to make up, a significant proportion of the NHS workforce. NHS bordering, and welfare bordering generally, can therefore be read as a continuation of imperial extraction, exploiting the wealth and labour of the former Empire while limiting former imperial subjects’ access to services (El-Enany, 2020; Medien, 2021; 2022). Additionally, Cassidy (2018) locates both visa checks and the introduction of the IHS in a wider project to maximise the economic benefits of tourism and migration to the UK. Hospital wards become the site of ‘scene[s] of exclusion’ (De Genova, 2013: cited in Cassidy, 2018), as overseas visitors officers^v^, acting under this logic of maximising the economic benefits of migration, exclude patients wrongly perceived as ineligible for care. These racialised dynamics of economic exploitation and exclusion, and the link between NHS bordering and citizenship and belonging more broadly, are critical in understanding the specific ‘situatedness’ of the NHS as a bordering scape.

## Methodology

This paper is based on critical discourse analysis of policy documents and Parliamentary debates around NHS bordering within the 2014 Immigration Act, undertaken as part of a Wellcome-funded^vi^ project exploring migrant women’s experiences of maternity care in the north of England. Unusually, there was no white paper or green paper associated with the 2014 Immigration Act. My analysis focuses instead on research and consultations undertaken or commissioned by the UK government in order to legitimate NHS bordering and to outline the possible forms, and potential targets, of these bordering processes. These documents include the public consultation about proposed changes published by the Home Office (2013a) and the Department of Health (2013a), as well as both institutions’ published responses to these consultations (Department of Health, 2013b; Home Office, 2013b). All of these documents address the question of who should be charged for NHS care; what services should be charged for (e.g. Primary and/or Secondary Care); and whether to introduce the IHS. Also analysed was the Equality Analysis^vii^ of the proposed changes undertaken by both the Home Office (included as part of (Home Office, 2013b)) and the Department of Health (2013c); as well as the Quantitative and Qualitative Research Reports commissioned by the Department of Health (Creative Research, 2013; Prederi, 2013). I additionally undertook critical discourse analysis of Hansard transcripts of Parliamentary debates around NHS bordering in the 2014 Immigration Act, to allow data triangulation. In combination, these documents and debates allow an understanding of government arguments for NHS bordering; the construction of migrants within these arguments; and of the wider hegemonic, and counter-hegemonic, discourses around NHS bordering. A full list of analysed documents can be found in Appendix One.

Critical Discourse Analysis understands discourse as both the product of social practices, and as socially constitutive; ‘the discursive event is shaped by situations, institutions, and social structures but it also shapes them’ (Fairclough et al., 2013: 80; see also Reisigl and Wodak, 2001). While there is no ‘standard’ CDA methodology (Fairclough et al., 2013), it is widely agreed among practitioners that it should be ‘problem-oriented’; and critical, illuminating the operation of power through discourse, revealing internal inconsistencies and contradictions within particular discursive formations, and uncovering how particular texts or speech acts persuade or manipulate their audiences (Fairclough et al., 2013; Wodak, 2001; Wodak, 2013).

For this paper, I drew on the Discourse Historical Approach pioneered by Wodak (c.f. Reisigl and Wodak, 2001; Wodak, 2001), as well as on Van Leeuwen’s (2008) scholarship on legitimation strategies. The Discourse Historical Approach (DHA) emphasises the importance of the socio-political and historical context in the analysis of discourse. Wodak (2001: 67) identifies four levels of relevant context within the DHA approach: that of the ‘immediate, language or text internal co-text’; intratextual and interdiscursive relationships; the social/institutional/situational context (referred to by Wodak as ‘middle range theories’); and the ‘broader sociopolitical contexts…(‘grand’ theories).’ The historical aspect is particularly salient, given the considerable influence exerted by past debates and practices around citizenship and migration on discourses, practices, and policies today (El-Enany, 2020; Medien, 2021; 2022; Samantrai, 2002; Tyler, 2010). Moreover, the third level of context, that of the institution, is helpful in exploring the *situatedness* of NHS bordering policies and their relationship to discourses around reproduction.

In their work on racism and antisemitism, Reisigl and Wodak (2001: 45, p. 45) identify five discursive strategies used to construct and justify racist discrimination; for this paper, three are of interest:

1. *Referential* or *nominational* strategies, by which particular groups are constructed as ‘insiders’ or ‘outsiders’;
2. *Predicational* strategies, which ‘strategies aim either at labelling social actors more or less positively or negatively, deprecatorily or appreciatively.’ These cannot always be separated from *nominational* strategies, as constructing ‘outsider’ groups may involve ‘deprecatory or appreciative labelling’;
3. *Argumentation* strategies, or *topoi,* which serve to justify the exclusion of particular groups or actors.

Van Leeuwen (2008) provides a framework for analysing how NHS bordering, and its particular forms and targets, are justified. He identifies four categories of legitimation strategies: A*uthorization* (legitimation referencing individual, traditional, or legal authority); *moral evaluation* (‘reference to value systems); *rationalization* (‘legitimation by reference to goals…’); and *mythopoesis* (where legitimation is ‘conveyed through narratives whose outcomes reward legitimate actions and punish nonlegitimate actions’) (Van Leeuwen, 2008: 105-106).

In keeping with CDA’s ‘problem-oriented’ approach, I began my analysis of the documents and Hansard by posing the following questions:

(How) are migrants constructed as a problem for the NHS? What key argumentation and legitimation strategies are used? Are any particular groups of migrants singled out?
(How) are *pregnant* migrants constructed as a problem for the NHS? What key argumentation and legitimation strategies are used? Are any particular groups of migrants singled out?

The policy documents were initially read carefully, with a view to answering these questions. A first round of analysis was conducted by hand, identifying key discursive and legitimation strategies and intertextual and interdiscursive relationships. A sample of the Hansard was then read and coded by hand, both to check the rigour of the established codes and *topoi,* and to identify analytical gaps. These strategies and relationships were translated into a coding framework, and policy documents and Hansard transcripts were (re)coded using Nvivo. This supplemented the initial coding, allowing a more granular analysis, another opportunity to verify the appropriateness of codes, and the identification of commonalities and differences in discursive and legitimation strategies across the different documents analysed, as well as facilitating data management.

## The 2014 Immigration Act and the legitimation of NHS bordering

### A ‘fair contribution’

The argumentation and legitimation strategies deployed in policy documents and parliamentary debates around NHS bordering in the 2014 Immigration Act constitute the NHS as a bordering site. One of the key moral legitimation strategies deployed in the analysed documents and debates presents NHS bordering as necessary to ensure migrants are making a ‘fair contribution’ to the cost of NHS services:

we propose that temporary non-EEA migrants should pay a migrant health levy on entry to the country that would be a *fair contribution* for access to any NHS services that they may subsequently require during their stay

(Department of Health, 2013a: 7, emphasis mine)

As is often the case with moral legitimation strategies (Kronick and Rousseau, 2015; Van Leeuwen, 2008), the value being referenced – a need for ‘fairness’ – is not well elucidated. In part, the call for ‘fairness’ is underpinned by the *topoi* of ‘migrants as economic burden.’ Migrants are costly to the NHS, and it is ‘unfair’ to expect the ‘British taxpayer’ to bear these costs. The Home Office (2013a: 4) call for consultation states:

The current rules on accessing the NHS by migrants are extremely generous when compared with other countries; and these rules are not being strictly applied. This means that the taxpayer is meeting the healthcare costs of both large numbers of people who should not be here and “health tourists” who deliberately seek to exploit the current weakness in our charging arrangements in order to receive free healthcare to which they are not entitled.

The deployment of this *topoi* is consistent with the wider history of depicting migrants as a burden on the welfare state (Samantrai, 2002; Solomos, 2003). However, there is significant ambivalence around this *topoi* as migrants are *also* constructed as bringing economic benefits to the UK, especially in discussions around the IHS. Various policy documents state that, when deciding the cost of the levy, the government will consider the ‘competitive advantage of the UK as a destination for study’ (Home Office, 2013a: p. 16), and ensure that the levy does not dissuade economically productive migrants (Department of Health, 2013b; Home Office, 2013b). In Hansard, concerns are expressed by both MPs and members of the public giving evidence that the levy will be off-putting to international students especially (c.f. HL Deb 10 February 2014; Parliament. House of Commons, 2013d). Some MPs also suggest that migrants impacted by the IHS are *already* making a ‘fair contribution’ through paying taxes and bringing economic benefits to the UK:

Some of the people affected will be working in the NHS or contributing in a range of other ways, so it seems odd that, if somebody is paying their taxes into the public purse and has done so for a certain amount of time, we ask them for further money as well

(Parliament, House of Commons, 2013d, p. 280).

This ambivalence in the construction of migrants is consistent with Cassidy’s (2018) argument that the bordering regime within the NHS can be understood as a strategy to maximise the financial benefits of migration and tourism.

### From ‘ordinary resident’ to ‘temporary migrant’

The call for migrants to make a ‘fair contribution’ relies not only on this ambivalent economic rationale, however. The moral legitimation strategy is intertwined with a referential strategy naming migrants affected by the IHS as ‘temporary’ residents without a ‘permanent relationship’ to the UK (c.f. Department of Health, 2013b: 6). This points to the introduction of the IHS as a significant moment of re-bordering. Prior to the 2014 Immigration Act, access to free secondary care on the NHS was available to anyone deemed ‘ordinary resident’ in the UK, defined as non-EEA migrants ‘who come to the UK to work, or to study, or for family reasons for more than 6 months’ (Department of Health, 2013a: 6; see also Department of Health, 2013c). In arguing for the necessity of the IHS, it is repeatedly asserted in policy documents and Hansard that these migrants should *not* be considered ordinary resident, but as ‘temporary migrants.’ The Home Office (2013a: 6, emphasis mine) call for consultation states: ‘We are therefore consulting on a proposal to change the existing ‘ordinary residence’ test which governs free access to the NHS to exclude *temporary* non-EEA migrants’. Similarly, during the second reading in the House of Commons, then-Home Secretary Theresa May argues

Many *temporary* migrants are currently allowed free access to the NHS as if they were permanent residents. Such an approach is extremely generous, particularly compared with wider international practice.

(HC Deb 22 October 2013, emphasis mine).

Importantly, the category of ‘non-EEA temporary migrants’ also appears in the Research Reports commissioned by the Department of Health (Creative Research, 2013; Prederi, 2013). A group of migrants was thus *named* by the government, and *then* consultations and research were commissioned to determine how these migrants posed a ‘problem.’

The moral argument for a ‘fair contribution’ relies significantly on this referential strategy:

‘For temporary migrants there was also support for seeking a fair contribution towards the cost of their care until they have formed a permanent relationship with the UK’

(Department of Health, 2013b: 13).

Thus, migrants are supposed to pay a ‘fair contribution’ *because* they are now deemed temporary (as opposed to ordinarily resident); this referential strategy can also be observed in the quote from Theresa May above, arguing the NHS is ‘too generous’ to temporary migrants. Similarly, the Home Office (2013b: 9) consultation response justifies the IHS as follows:

Whilst we recognise that temporary migrants may also contribute to the economy, the tax paid by a temporary migrant who will be living in the UK only for a limited time, even a high net worth individual, will generally be less than that of a permanent resident worker over his/her lifetime. *In addition, their depth of connection to the UK is weaker than that of permanent migrants*

(emphasis mine).

Initially, the rationale for the levy is that ‘temporary migrants’ will have made less of an economic contribution to the UK economy. However, it is also suggested that a ‘temporary migrant’ will have less of a *connection* to the UK. Paying the health levy is thus posited as necessary not only to ensure migrants are making a ‘fair contribution’ but also as a consequence of their attributed emotional distance from the UK.

The Immigration Health Surcharge thus served to redefine who could be considered ‘at home’ in the UK; introducing the IHS rendered migrants on work, student, and family visas ‘temporary’ rather than ‘ordinary resident.’ Although welfare bordering often has domopolitical implications, it is rarely made this explicit. Access to the NHS is associated, through the IHS, with being ‘at home’ in the UK. This domopolitical deployment of the NHS takes on racial undertones within documents and debates, in keeping with the association between ‘Britishness’ and whiteness in bordering processes more broadly, and the tendency to conflate migrant and ‘ethnic minority.’ The introduction of the IHS created the category of ‘temporary *non-EEA* migrants’^viii^, a group racialised in the popular imagination as non-white (despite the large numbers of white non-EEA migrants from e.g. Canada and Australia) (Bhambra, 2018). This is reinforced by the Qualitative Research Report (Creative Research, 2013: 29) in particular, which associates the presence of ‘non-EEA temporary migrants’ with areas with ‘diverse local ethnic populations’. It is also asserted within the Report that British citizens are helping migrant family members engage in health tourism:

There were also anecdotes of family members coming to the UK on annual visits and using the occasion to have a check-up or access treatment.

(p. 29)

Again, this is associated with areas with ‘diverse local ethnic populations’; ethnic minority citizens are thus charged with undermining the ‘national home’ by supporting health tourism. Moreover, it suggests that citizens with non-British family – who are likely to have immigration backgrounds themselves – pose a threat to the NHS, calling into question their commitment to the UK.

## Pregnant migrants within NHS bordering

These legitimation and argumentation strategies interact with gendered and racialised discourses around reproduction in the construction of NHS bordering processes, and the production of pregnant migrants as a particular social location within these processes. Through a domopolitical lens, women’s role in reproducing the family home mirrors her role reproducing the ‘national home’ (Lonergan, 2018). The use of the IHS to define who is ‘at home’ serves to construct pregnant migrants affected by the levy, and their children, as ‘temporary’, and not British. Pregnant migrants therefore – whether short-term visitors or those on work, family, or student visas - are introducing an alien population. Limiting migrants’ use of NHS maternity services is necessary to discipline the arrival of ‘unwanted guests’ in the national home. Simultaneously, in giving birth to children, and building a family, a migrant woman is also building a home, suggesting that she, and her family *are* at home in the UK, regardless state assertions of temporariness. Associating access to the NHS with being ‘at home’ can paradoxically serve to strengthen these implicit claims – what could be more British than a baby born on ‘our NHS’^1^? This heightens anxieties about the prospect of migrant women reproducing the ‘national home’, while also undermining the use of the NHS to define this home. These insecurities are reflected in the Qualitative Research Report (Creative Research, 2013: 109) which explicitly suggests that non-EEA migrants may be ‘seeking to use the UK’s maternity services for reasons that they were not keen to share’, including

using the period following the birth and the fact that their child has been born in the UK to help in their application for leave to remain (having arrived too late to return home).

### The problem of ‘maternity tourism’

Pregnant migrants are consequently constituted as an especial ‘problem’ within the analysed debates and documents. Maternity services and pregnant migrants feature more prominently in policy documents than any other secondary care specialism or patient. A count was conducted of the frequency of words associated with particular secondary care specialisms (e.g. oncology and cancer are grouped together)^ix^ within analysed documents^x^. The results are shown in Table One. Although most documents may contain only a few mentions of maternity services, other specialisms are mentioned far more rarely.

Furthermore, the impact of migrants on maternity care is a key *topoi* deployed to legitimate NHS bordering. The original Home Office(2013a) call for consultation states:

Within the NHS, there is considerable anecdotal evidence of abuse, relating to *maternity services* and treatment for acute conditions including cancer and renal services, as well as access to other routine elective procedures

(p. 22, emphasis mine).

Similarly, the Department of Health (2013b: 9) response to the consultation states, for example:

EEA temporary residents and their families are often felt to be having a significant impact locally, on a range of services *including maternity*, because of their numbers

(emphasis mine).

‘Maternity tourists’ are also named within Parliamentary Debates, especially in the House of Commons, as a key reason a stricter charging regime is needed; for example, giving evidence in Committee, Jacqueline Bishop, the co-chair of the Overseas Visitors Advisory Group of the NHS stated:

In my hospital, I do not think we have one particular area that people come for. Maternity is rife throughout. Anyone who says that they do not have overseas visitors in maternity either are not doing their job properly or just have their head in the sand

(Parliament. House of Commons, 2013, 8).

Moreover, within the Qualitative Research Report especially, ‘maternity tourism’ is at times conflated with *any* use of the NHS by pregnant migrants. Discussing the impact of ‘Visitors who Fly in and Fly Out’ (i.e. intentional health tourists), the Report uses 13 quotes by NHS staff providing examples of such patients. Five involved antenatal or maternity care, and of these, three were about people who were ordinary resident at the time the report was written, and therefore legally entitled to maternity care. Both of the examples under the heading of ‘patients on student visas who are not attending college’ were of pregnant migrants; one states:

Yeah, I think there're a number of people coming on student visas who aren't actually a student. And if we asked them to provide evidence that they are actually studying, regardless of the NHS number, if we were doing it legitimately and properly, I think you would find there is a lot of people using maternity services while on a student visa and aren't actually studying. So they've used the visa to get over here and the entitlement for free treatment, but the reality is they've come to have the child

(p. 41).

Similarly, discussing the impact of ‘visitors who fly in and fly out’ on primary care, a CCG representative is quoted:

Although I do have a background suspicion about the numbers who, looking at the names of our patients who are currently pregnant, there’s a very heavy non-English name dominance. So whether there is some maternity tourism and ‘come and get a job so I can have my babies and then go back home’, I don't know, but I wouldn’t be at all surprised

(p. 42).

Individuals on work and student visas were at the time of the report ‘ordinary resident’; ‘maternity tourism’ is thus conflated migrants having babies in NHS maternity services. The implication in both quotes is that there is something inherently illicit about *any* pregnant migrants using the NHS. While this claim is not explicitly made in other documents analysed, both the Department of Health (2013b: 29) response to consultation, and its Equality Analysis (2013c) cite the Qualitative Research Report as demonstrating that ‘deliberate maternity health tourism through the short-term visit entry is a problem.’ This validates the expansive view of ‘maternity tourism’ adopted by the Report, reinforcing the construction of pregnant migrants’ use of maternity services as inherently illicit.

The racialisation of the ‘national home’ present in the domopolitical deployment of the NHS is also visible in the ‘problematisation’ of pregnant migrants. Although EEA migrants are also cited as having an impact on maternity services, the ‘maternity tourist’ in particular – a figure who is not only impacting NHS services, but doing so illicitly - is often named as West African, in both Hansard and in the Qualitative Research Report (Creative Research, 2013). Discussing ‘maternity tourism’ before the House of Commons committee, Professor J. Meiron Thomas states

If you go to the obstetrics and gynaecology department at Guy’s and St Thomas’ across the river, they will talk about the *Lagos shuttle,* for example

(Parliament. House of Commons, 2013a, p. 14, emphasis mine).

This is in keeping with the wider racialised underpinnings of the original emergence of NHS bordering in 1982, as well as anxieties around migrant women’s ability to reproduce the nation-state. The association between Britishness and whiteness is such that Black migrant women are constructed as a particular threat to the reproductive future of the nation.

### Pregnant migrants as disposable economic burdens

The ‘problematization’ of pregnant migrants is additionally underpinned by the interaction of (neo)liberal discourses of citizenship which construct reproductive labour as economically non-productive (Bakker, 2007) with the NHS as a bordering site, and the strategy of maximising the economic benefits of migration (Cassidy, 2018). Children born to the migrants targeted by the 2014 immigration act are not automatically entitled to UK citizenship^xi^. Providing maternity services to these pregnant migrants thus involves investing state resources in an activity (reproduction) deemed economically non-productive, and that does not reproduce the ‘national home’. Consequently, the ambivalence around the economic benefits of NHS bordering is absent from depictions of pregnant migrants. Pregnant migrants are exclusively constructed as economically burdensome. Within both Hansard and policy documents, for example, students are frequently named as ‘desirable’ migrants; yet, as noted above pregnant migrants on student visas are portrayed as ‘maternity tourists’.

Pregnant migrants also appear in both documents and debates as an especially vulnerable group; perversely, however, this serves to reinforce the predicational strategy naming them as economically burdensome. A key moral legitimation strategy deployed throughout the analysed documents involves insisting that the ‘most vulnerable’ will be exempt from NHS bordering:

Vulnerable groups such as asylum seekers, refugees, humanitarian protection cases and victims of human trafficking will also continue to have free access to the NHS in line with our international commitments, and will not be subject to the surcharge. Certain vulnerable groups, including children in local authority care, will not be required to pay a surcharge, and will continue to have free access to the NHS

(Department of Health, 2013b: 23)

Pregnant migrants are not one of the groups named as exempt in the above quote; but they are routinely portrayed as vulnerable. During the Second Reading of the Bill in the House of Lords, for example, Lord Patel argued:

Charges at the point of care create risks that women will not present to the NHS, will present late in pregnancy or will be denied access because of their inability to pay. This prevents midwives and doctors from giving the appropriate health advice and treatment early in pregnancy. It cannot be right to include pregnant women

(HL Deb 10 February 2014).

Moreover, the government responses to both the Home Office consultation and the Department of Health consultation (Department of Health, 2013b; Home Office, 2013b) acknowledge respondents suggesting that pregnant migrants could be harmed by the proposed policy changes. The Department of Health (2013b: 29) response states:

[T] the risks to the health of both the mother and baby if refused or deterred by the need to pay are significant… However, our independent research confirms that deliberate maternity health tourism through the short-term visit entry is a problem, and this could only increase, potentially significantly, if services were provided free of charge. We therefore shall not be introducing any new exemptions from charging for maternity services.

However, pregnant migrants are *not* exempt from charging. In their analysis of Canadian parliamentary debates around the mandatory detention of migrant children, Kronick & Rousseau (2015) note that the construction of migrant children as worthy of compassion implicitly constructs adult migrants as unworthy. A similar dynamic can be observed with regard to NHS bordering; pregnant migrants are *so costly* that their vulnerability must be overlooked. Yet, strikingly, the Department of Health response also states that ‘maternity tourism’ might be included in a wider category of ‘health tourism’ costing the NHS somewhere between £20m and £100m per year – or between 0.018 % and 0.089% of the 2013/2014 NHS budget of £112bn (Lafond, 2015). In this context, the insistence that ‘maternity tourism’ requires charging for maternity services, regardless of the risks to migrant mothers’ health, constructs pregnant migrants as ‘disposable’: ‘unnecessary burden on state coffers…consigned to fend for themselves’ (Giroux, 2006: 174). Any amount of money spent on maternity services for pregnant migrants is too much, regardless of the potential harm. This rendering pregnant migrants disposable becomes particularly visible in light of the 2019 MBRRACE-UK report on maternal mortality and morbidity, which linked three maternal deaths to visa checks within the NHS (Knight et al., 2019). Despite this, as of writing, the government has refused to exempt pregnant migrants from charges.

## Conclusion

This paper contributes to conceptualisations of bordering processes as situated and intersectional (Yuval-Davis et al., 2018; 2019), analysing NHS bordering in the 2014 Immigration Act to explore how these processes are informed and shaped by discourses dealing with the reproduction of the nation-state. Policies around migration and those governing reproduction are both informed by anxieties around the reproductive futures of the nation-state (Ross, 2006). With the multiplication and proliferation of internal bordering processes and sites, however, it is necessary to reconceptualise our understanding of the relationship between immigration policies and reproduction. This paper argues that discourses and anxieties around the reproduction of the nation-state, which are heavily racialised and gendered, interact with the specificities of individual bordering sites in shaping both bordering processes embedded in these sites, and the production of different social locations within these processes.

This paper also makes important empirical contributions by analysing the way in which pregnant migrants are produced as a ‘problem’ within policy documents and debates around NHS bordering in the 2014 Immigration Act. These documents and debates deploy a moral legitimation strategy that calls for migrants to make a ‘fair contribution’ for NHS services. This strategy is underpinned by conflicting depictions of migrants as economically burdensome, and economically beneficial. It is further intertwined with a referential strategy naming migrants on work, family, and student visas as ‘temporary’ rather than ‘ordinary resident.’ This serves to redefine who is considered ‘at home’ in the UK, and implicates the NHS in the construction of the ‘national home.’ In relationship with (neo)liberal discourses around reproduction, this results in the construction of pregnant migrants as both a key target of, and reason for, NHS bordering, and as disposable economic burdens. Babies born to pregnant migrants are ‘undesirable aliens’, while pregnant migrants are economically nonproductive and therefore unable to make a ‘fair contribution’ to the NHS. Yet, through a domopolitical lens, women’s role in reproducing the ‘national home’ is paralleled by their role in reproducing the ‘private home’. In having children in the UK, pregnant migrants can be read as building a home in the UK, contesting their ‘temporariness’, and subverting the association between the NHS and the ‘national home.’ Maintaining the integrity of that ‘home’, and the usefulness of the NHS in defining who can be ‘at home’, requires severely restricting pregnant migrants’ access to the NHS – even where it is acknowledged this could lead to severe harm. Pregnant migrants are thus produced as a particular social location, and an especial problem, through the interaction of discourses around reproduction with the specificities of the NHS as a bordering site.

This paper focuses on one specific bordering site; further research is therefore required to better understand the way discourses around reproduction interact with bordering processes embedded in other sites. Additionally, the multiplication and proliferation of internal bordering processes is not unique to the UK (Mezzadra and Neilson, 2013); nor are anxieties about national reproductive futures and the ability of migrant women to ‘reproduce’ the nation-state. Luibhéid (2013) discusses, for example, the way in which anxieties around pregnant asylum-seekers and their Irish-born babies contributed to the abolition of *jus soli* citizenship in the Republic of Ireland. Marchesi (2012) and Goldade (2011) both analyse the stigmatisation of migrant women’s fertility in Italy and Costa Rica respectively. Discourses around the reproduction of the nation-state are both nationally and locally situated, and will therefore inform the production of bordering processes in nationally and locally specific ways. Further research is required to grasp the way in which anxieties and discourses around reproduction inform the construction of these bordering processes, and the implications for migrants, especially racialised women and birthing people. The current political environment makes this research especially necessary; Erel (2018) has noted the growing focus on family life and reproduction among both far-right and right-wing politicians in Europe, and the targeting of racialised migrant families in immigration policy.

## Supplementary Material

Appendix One

## Figures and Tables

**Table One:** Word count related to secondary care specialisms in analysed documents

Document	Maternity	Oncology	Renal	Cardiology	HIV	Orthopaedics
Creative Research (2013)	104	21	54	8	7	6
Department of Health (2013a)	0	0	0	0	0	0
Department of Health (2013b)	19	1	0	0	3	0
Home Office (2013a)	3	1	1	0	0	0
Home Office (2013b)	4	0	0	0	3	0
**Total**	130	23	55	8	13	6

## Endnotes

i It should be noted that under devolution, each of the nations that makes up the UK – England, Wales, Scotland, and Northern Ireland –has its own NHS (Bevan et al., 2014). The 2014 Immigration Act, including all of its provisions involving the NHS, applies across the UK; however, individual countries can and do exercise discretion in the implementation of some health charging policies. Nonetheless, for the safe of simplicity, this paper refers to a single, UK-wide, NHS, reflecting its entanglement in bordering processes.

ii
https://bills.parliament.uk/bills/1298/stages

iii
https://www.legislation.gov.uk/uksi/2015/792/contents/made

iv Whereby one automatically becomes a citizen if one is born within a national territory, regardless of one’s parents’ nationality.

v These are NHS staff whose job it is to determine which patients may be charged for care, and to ensure they are charged

vi Grant number 209915/Z/17/Z

vii Undertaken as a requirement under the Equality Act 2010 to ensure that the law does not discriminate against individuals on the grounds of age, disability, gender reassignment, marriage and civil partnership, pregnancy and maternity, race, religion or belief, sex, or sexual orientation (Department of Health, 2013c)

viii Post-Brexit, however, all migrants, regardless of their country of origin, are subject to the IHS

ix Accident and Emergency was omitted because there was an ongoing debate about whether these services should remain free of charge regardless of immigration status

x The Department of Health (2013c) Equality Analysis and the Quantitative Research Report (Prederi, 2013) were omitted because maternity was mentioned frequently in both documents for methodological reasons; i.e. the Equality Analysis addressed maternity as a protected characteristic, and the Report followed the model used by the Department of Health accounts in treating maternity separately from other secondary specialisms (p. 18). Similarly, references to pregnancy and maternity in other documents were omitted where mentioned solely as a protected characteristic.

xi Unless their other parent is a UK citizen or has Indefinite Leave to Remain
