# Supplementary material for: Reproduction and the expanding border: pregnant migrants as a ‘problem’ in the 2014 Immigration Act
Source: Sociology. Author manuscript; Available in PMC 2024 Feb 1. (PMC10824606; doi:10.1177/00380385231157987)
Supplement: Appendix One [file EMS174838-supplement-Appendix_One.pdf]

Online Appendix: Documents Analysed

**Government Policy Documents**	**Title & Bibliographical Information**	**Description**
	Creative Research (2013) *Qualitative Assessment of Visitor and Migrant use of the NHS in England: Observations from the Front Line.* London	Research commissioned by the Department of Health
	Prederi (2013) *Quantitative Assessment of Visitor and Migrant Use of the NHS in England: Exploring the Data* London	Research commissioned by the Department of Health
	Department of Health (2013a) *Sustaining Services, Ensuring Fairness: A consultation on migrant access and their financial contribution to NHS provision in England*. London	Call for consultation responses
	Department of Health (2013b) *Sustaining services, ensuring fairness: government response to the consultation on migrant access and financial contribution to NHS provision in England*. London	Government response to the consultation
	Department of Health (2013c) *Sustaining services, ensuring fairness: Government response to the consultation on migrant access and financial contribution to NHS provision in England. Equality Analysis*. London	Equality analysis of proposed changes
	Home Office (2013a) *Controlling Immigration – Regulating Migrant Access to Health Services in the UK. Consultation Document*. London	Home Office call for consultation responses
	Home Office (2013b) *Controlling Immigration – Regulating Migrant Access to Health Services in the UK. Results of the Public Consultation*. London	Results of the consultation and Home Office Response
**Hansard**	HC Deb (22 October 2013) vol. 569, cols 156-257. Available at: https://hansard.parliament.uk/commons/2013-10-22/debates/13102262000002/ImmigrationBill (Accessed 4 February 2022)	Second Reading of the Bill, House of Commons
	Parliament. House of Commons (2013a) *Public Bill Committee. Immigration Bill.* Available at: https://publications.parliament.uk/pa/cm201314/cmpublic/immigration/131029/am/131029s01.pdf. (Accessed 4 February 2022)	Morning session of the Public Bill Committee for the Immigration Bill. 29.10.13
	Parliament. House of Commons (2013b) *Public Bill Committee. Immigration Bill.* Available at: https://publications.parliament.uk/pa/cm201314/cmpublic/immigration/131029/pm/131029s01.pdf. (Accessed 4 February 2022)	Afternoon session of the Public Bill Committee for the Immigration Bill. 29.10.13
	Parliament. House of Commons (2013c) *Public Bill Committee. Immigration Bill.* Available at: https://publications.parliament.uk/pa/cm201314/cmpublic/	Morning session of the Public Bill Committee for

	immigration/131031/am/131031s01.pdf (Accessed 4 February 2022)	the Immigration Bill. 31.10.13
	Parliament. House of Commons (2013d) *Public Bill Committee. Immigration Bill.* Available at: https://publications.parliament.uk/pa/cm201314/cmpublic/ immigration/131107/pm/131107s01.pdf (Accessed 4 February 2022)	Afternoon session of the Public Bill Committee for the Immigration Bill. 07.11.13
	Parliament. House of Commons (2013e) *Public Bill Committee. Immigration Bill*. Available at: https://publications.parliament.uk/pa/cm201314/cmpublic/ immigration/131112/am/131112s01.pdf (Accessed 4 February 2022)	Morning session of the Public Bill Committee for the Immigration Bill. 12.11.13
	HL Deb (10 February 2014). vol. 752, cols 415-528. Available at: https://publications.parliament.uk/pa/ld201314/ldhansrd/text/140210-0001.htm#1402104000322 (Accessed 4 February 2022)	Second Reading of the Bill, House of Lords
	HL Deb (10 March 2014). vol 752, cols 1551-1579; Available at: https://publications.parliament.uk/pa/ld201314/ldhansrd/ text/140310-0001.htm#1403105000403 (Accessed 4 February 2022)	House of Lords Immigration Bill Committee 10.03.14
	HL Deb (12 March 2014). vol. 752, cols 1802-1848; Available at: https://publications.parliament.uk/pa/ld201314/ldhansrd/ text/140312-0002.htm (Accessed 7 February 2022)	House of Lords Immigration Bill Committee 12.03.14
	HL Deb (3 April 2014). vol 753, cols 1096-1102; Available at: https://publications.parliament.uk/pa/ld201314/ldhansrd/ text/140403-0002.htm#14040353000571 (Accessed 7 February 2022)	House of Lords Immigration Bill Report 03.04.14
	HC Deb (30 January 2014) vol 573, cols 1025-1130; Available at: https://publications.parliament.uk/pa/cm201314/cmhansrd/ cm140130/debtext/140130-0002.htm#14013058000001 (Accessed 7 February 2022)	Third Reading of the Bill, House of Commons
	HL Deb (06 May 2014) vol 753, cols 1402-1421; Available at: https://publications.parliament.uk/pa/ld201314/ldhansrd/ text/140506-0001.htm#14050619000594 (Accessed 7 February 2022)	Third Reading of the Bill, House of Lords
